# Clinical and neuropsychological features associated with progression in subjective cognitive decline

**DOI:** 10.3389/fnagi.2025.1680762

**Published:** 2025-11-26

**Authors:** Rafael Villino-Rodríguez, Mirla M. Ríos-Rivera, Genoveva Montoya-Murillo, Jonathan Patricio Baldera, Salomón Salazar-Londoño, María Cruz Rodríguez-Oroz, Miguel Germán Borda, Adolfo Jiménez-Huete, Mario Riverol

**Affiliations:** 1 Department of Neurology, Clínica Universidad de Navarra, Pamplona, Spain; 2 Department of Neurology, Hospital Universitario HM, Sanchinarro, Madrid, Spain; 3 School of Medicine, Universidad Autónoma de Chiriquí, David, Panama; 4 Centre for Age-Related Medicine (SESAM), Stavanger University Hospital, Stavanger, Norway; 5 Semillero de Neurociencias y Envejecimiento, Ageing Institute, Medical School, Pontificia Universidad Javeriana, Bogotá, Colombia

**Keywords:** subjective cognitive decline, Alzheimer’s disease, mild cognitive impairment, neuropsychological assessment, disease progression

## Abstract

**Background and objectives:**

Subjective Cognitive Decline (SCD) is recognized as an early indicator of neurodegeneration, yet factors that predict its progression to mild cognitive impairment (MCI) or dementia remains not fully understood. In this study, we aim to identify clinical and neuropsychological features associated with the progression of SCD.

**Methods:**

450 persons with SCD were included, consisting in 319 non progressors (SCDnp) and 131 progressors (SCDp) to MCI or dementia due to AD. The study was conducted at the Clínica Universidad de Navarra Memory Clinic between 2001 and 2017. We included data on medical interviews and neuropsychological evaluations. Differences between SCDnp and SCDp were assessed and, to evaluate the association between exposure variables and progression in time, proportional-hazards Cox models were applied. In addition to the exposure variables, the models were adjusted for age, sex, and years of education.

**Results:**

At baseline, SCDp were older, had a higher prevalence of hypertension and hypercholesterolemia and had worst performance on tests related to processing speed, verbal fluency, visual memory, verbal memory, and executive functioning. Factors associated with progression at follow-up were lower scores in some cognitive tests: MMSE, TMT-B, and the CERAD regarding trial 1 of immediate recall, trial 2 of immediate recall, trial 3 of immediate recall and the delay recall score.

**Discussion:**

Lower scores on global cognition, executive functioning and verbal memory tests were predictors of progression to MCI or dementia in patients with SCD. These findings underscore the importance of nuances in neuropsychological evaluation, even with a normal score, for detecting high-risk individuals for early intervention.

## 1 Introduction

Dementia is one of the most disabling diseases in older adults, imposing a significant societal burden due to its high frequency and costs (Scheltens et al., 2016). In the natural evolution of cognitive impairment, such as in Alzheimer’s disease (AD), patients progress through three phases: preclinical, where disease mechanisms begin without noticeable cognitive decline; prodromal, marked by mild cognitive impairment (MCI); and dementia, where cognitive impairment significantly affects daily life functioning (Jack et al., 2024). However, some individuals in the preclinical phase may experience mild cognitive symptoms, or report a complaint about their cognitive performance, while still performing normally on cognitive tests. This condition is now referred to as subjective cognitive decline (SCD), and its distinction with MCI relies on neuropsychological evaluations (Jessen et al., 2020).

SCD is a common condition in aging, affecting 25–50% of individuals aged 65 years or older (Jessen et al., 2014). It has been described that it can emerge even up to 15 years before an MCI diagnosis (Jessen et al., 2014; Molinuevo et al., 2017). Moreover, while often linked to neurodegenerative disorders, it can also result from normal aging, systemic illnesses, psychiatric conditions and non-neurodegenerative neurological disorders (Jessen et al., 2014; Reisberg et al., 2008). Thus, SCD is not always a direct indicator of preclinical AD (Jessen et al., 2014), or of another neurodegenerative disease.

Various factors can contribute to the development of SCD, with depressive symptoms being especially important (Liew, 2019). Studies suggest that having SCD and depression predicts cognitive decline and correlates with increased amyloid pathology, highlighting its potential as a longitudinal risk marker (Kleineidam et al., 2023). However, it is not always easy to determine whether SCD is solely present in the context of depression as a primary psychiatric disorder, or concomitantly as a neuropsychiatric symptom in an underlying neurodegenerative process.

Evidence also suggests that other clinical factors, such as cardiovascular risk, may be associated with the onset of SCD. However, the role of these factors in the progression to MCI or AD dementia remains unclear. While some studies indicate a possible link, the findings are often inconsistent (Dufouil et al., 2005).

Considering the relevance of neuropsychological performance and the initial clinical evaluation in SCD diagnosis, in this study we aim to investigate the factors related with progression from SCD to MCI or dementia.

## 2 Methods

An observational longitudinal cohort study was designed, and the STROBE checklist for cohort studies was followed. The initial sample included 1,013 patients who were evaluated at the memory clinic of Clínica Universidad de Navarra between 2001 and 2017. Of these, 945 individuals with SCD—defined as self-reported memory complaints despite normal performance on neuropsychological tests—were initially considered. The diagnosis of SCD was based on the Jessen SCD-I criteria. The SCD-plus criteria were not applied because the necessary data to fulfill those criteria, apart from age, were not available (Jessen et al., 2014). Differences between participants who were not included in the analysis due to lack of follow-up and those who completed follow-up are presented in Supplementary material.

To determine the normality of cognitive status, the scores obtained from the tests were adjusted for age, sex, and education level. This yields a cutoff point of ≥7 in scaled scores, according to the standardization for the Spanish population, based on the ‘Neuronorma’ project (Rocca et al., 2012; Lopez Miquel and Agustí, 2011; Casals-Coll et al., 2013; Peña-Casanova et al., 2009; Peña-Casanova et al., 2009; Peña-Casanova et al., 2009). Normal cognitive function was defined as performance exceeding −1.5 standard deviations (SD) from the age-, sex-, and education-adjusted normal range on all tests (Molinuevo et al., 2017; Rocca et al., 2012; Lopez Miquel and Agustí, 2011; Casals-Coll et al., 2013; Peña-Casanova et al., 2009; Peña-Casanova et al., 2009; Peña-Casanova et al., 2009).

All participants were evaluated by a behavioral neurologist. The initial assessment included a medical and history review, an interview with a family member or friend, and a general and neurological examination. All participants underwent laboratory tests (i.e., full blood count, biochemistry, vitamin B12, serum folate, glucose, lipids, syphilis serology and thyroid function), neuropsychological assessment, and brain magnetic resonance imaging (MRI). Data from patients were reviewed in a multidisciplinary consensus meeting to determine a clinical diagnosis.

The exclusion criteria for the present study were: having MCI or dementia (McKhann et al., 2011; Albert et al., 2011), significant neurological or systemic illness that could lead to cognitive impairment, present or past major psychiatric disorders (e.g., schizophrenia, major depression, and bipolar disorder), a history of alcohol or substance abuse, notable MRI abnormalities (e.g., brain tumors, large cerebral infarct, and bleeding), and a previous head trauma resulting in loss of consciousness.

Of the 945 participants with SCD, 450 were followed up at our clinic and completed one or more follow-up visits through January 2020. Follow-up was voluntary, with patients typically scheduled for annual evaluations. Each visit included an assessment by a neurologist and a comprehensive neuropsychological evaluation to determine clinical progression. Follow-up concluded when a diagnosis of MCI or dementia was made.

Among the 450 patients who were followed, 131 progressed to MCI or dementia (AD dementia = 16 patients, non-AD dementia = 1 patient [Vascular dementia]) and were classified as SCD progressors (SCDp). In contrast, 319 remained clinically stable and were classified as SCD non-progressors (SCDnp) during the follow-up (see Supplementary material). The diagnoses of MCI and AD were based on the clinical criteria established by the National Institute on Aging and the Alzheimer’s Association (NIA-AA) in 2011 (McKhann et al., 2011; Albert et al., 2011).

This study was approved by the Ethics Research Committee of the Universidad of Navarra. Written informed consent was obtained from all participants.

### 2.1 Measures

Demographic variables included age of SCD diagnosis, age of MCI or dementia diagnosis, sex and years of education. Based on self-report, report by a family member or by clinical records, registered medical conditions included arterial hypertension, diabetes mellitus, hypercholesterolemia, cardiovascular disease (e.g., hearth failure, acute myocardial infarction), cerebrovascular disease (including ischemic and hemorrhagic strokes) and smoking.

All subjects underwent a neuropsychological assessment to evaluate cognitive status at baseline using a comprehensive test battery that evaluated the following domains: global cognitive function [Mini-Mental State Examination (MMSE)]; depression [Geriatric Depression Scale (GDS) with 30 items]; episodic verbal memory (word list learning, recall and recognition) and episodic visual memory (figure recall) based on the [The Consortium to Establish a Registry for Alzheimer’s Disease Word List Memory Task (CERAD)]; processing speed [Trail Making Test (TMT) parts A]; executive function [Trail Making Test (TMT) part B], phonetic fluency (words with letter p), cognitive interference [The Stroop Colour and Word Test (SCWT)]; and language [animal categories and the Boston naming test (BNT)] (Rocca et al., 2012; Lopez Miquel and Agustí, 2011; Casals-Coll et al., 2013; Peña-Casanova et al., 2009; Peña-Casanova et al., 2009; Peña-Casanova et al., 2009).

Interindividual differences are supported by a standardized medical and cognitive assessment, improving reliability. As mentioned, the normality of cognitive tests was based on already stablished cutoff points for the Spanish population. All patients received the same protocolized assessment by a behavioral neurologist, and the same diagnostic criteria was applied. All the patients come from the same cohort and were evaluated at the same center.

### 2.2 Statistical analysis

An initial description of the cohort was produced by calculating means ± standard deviations (SD) for continuous variables and frequencies (percentages) for categorical variables. The comparative analyses between individuals with subjective cognitive decline and healthy controls are presented in Supplementary material. Differences between the subjective cognitive decline (SCD) as a progressors (SCDp) and non progressors (SCDnp) group were assessed with Student’s *t*-test for both means and proportions. Also, differences between the subjective cognitive decline (SCD) group and the control group were assessed with Welch’s *t*-test for both means and proportions, given the marked imbalance in sample size (945 vs. 68 subjects) and the likelihood of unequal variances (see Supplementary material).

To evaluate the association between the clinical and neuropsychological variables of interest and the risk of progressing from SCD to more advanced cognitive impairment, proportional-hazards Cox models were applied. The dependent variable was the time (months) elapsed from the baseline assessment to the date of clinical progression (event). In addition to the exposure variables, the models were adjusted for age, sex, and years of education. Cognitive test scores were standardized (age-adjusted z-scores) to facilitate comparability across domains, and hazard ratios were expressed per one standard deviation (1-SD) increase in the corresponding variable. The proportional-hazards assumption was verified with the global Schoenfeld residuals test, which showed no significant violations (*p* > 0.05). Results are reported as hazard ratios (HRs) with their corresponding 95% confidence intervals (95% CI) and *p*-values; Kaplan–Meier curves with 95% confidence bands and log-rank tests were constructed for selected exposure variables. For those exposure variables, the values were dichotomized based on the first quartile of their distribution in the sample, classifying participants with scores ≤ Q1 versus those with scores > Q1. A sensitivity analysis was conducted for both baseline demographic and clinical characteristics, comparing included and excluded participants to assess potential selection bias. All analyses were performed in R Studio version 4.3.1, and statistical significance was set at *α* = 0.05 and the Benjamini-Hochberg method was used to adjust for multiple comparisons.

## 3 Results

At baseline, SCDp were older and had a higher prevalence of hypertension and hypercholesterolemia. Regarding the neuropsychological analysis, SCDp had worst performance on tests related with processing speed (TMT-A), verbal fluency (animals), visual memory (figure recall), verbal memory (CERAD: immediate recall, delay recall, recognition score) and executive functioning (Stroop total and TMT-B). After adjusting the scores for age, the test related to visual memory and one of the tests assessing executive function (Stroop total) no longer reached statistical significance.

Descriptive characteristics are displayed in Table 1.

**Table 1:** Clinical and neuropsychological differences between groups at baseline.

Variables	Overall	SCDnp	SCDp	*p* - value
	(*n* = 450)	(*n* = 319)	(*n* = 131)	
Age, mean (SD)	65.2 (10.2)	63.1 (10.3)	70.1 (8.04)	**< 0.001**
Sex (men, %)	240 (53.3%)	175 (54.9%)	65 (49.6%)	0.313
Education in years, mean (SD)	12.8 (4.23)	12.8 (4.23)	12.8 (4.24)	0.968
Hypertension, (%)	223 (49.6%)	143 (44.8%)	80 (61.1%)	**0.002**
Diabetes mellitus, (%)	78 (17.3%)	50 (15.7%)	28 (21.4%)	0.147
Hypercholesterolemia, (%)	263 (58.4%)	173 (54.2%)	90 (68.7%)	**0.005**
Smoking, (%)	144 (32.0%)	104 (32.6%)	40 (30.5%)	0.670
Cerebrovascular disease, (%)	40 (8.9%)	24 (7.5%)	16 (12.2%)	0.113
Cardiovascular disease, (%)	41 (9.1%)	29 (9.1%)	12 (9.2%)	0.982
GDS, mean (SD)	9.10 (5.69)	9.36 (5.78)	8.50 (5.46)	0.149
MMSE, mean (SD)	28.5 (1.57)	28.5 (1.50)	28.4 (1.74)	0.400
Processing speed				
TMT-A, seconds, mean (SD)	46.1 (19.6)	43.9 (18.5)	51.4 (21.0)	**< 0.001**
TMT-A, seconds (age-adjusted z score), mean (SD)	0.00 (1.00)	−0.068 (0.95)	0.166 (1.09)	**0.026**
Verbal fluency				
BNT, mean (SD)	50.9 (6.75)	51.3 (7.32)	50.0 (5.06)	0.067
BNT (age-adjusted z score), mean (SD)	0.00 (1.00)	0.0517 (1.09)	−0.121 (0.748)	0.106
Animals, score, mean (SD)	16.6 (5.20)	17.2 (5.14)	15.3 (5.13)	**0.001**
Animals (age-adjusted z score), mean (SD)	0.00 (1.00)	0.0707 (1.00)	−0.168 (0.978)	**0.023**
Constructional ability				
Figure copy, mean (SD)	9.98 (0.309)	9.99 (0.260)	9.95 (0.405)	0.199
Figure copy (age-adjusted z score), mean (SD)	0.00 (1.00)	0.0370 (0.840)	−0.0909 (1.31)	0.236
Visual memory				
Figure recall, mean (SD)	6.53 (3.85)	6.80 (3.81)	5.85 (3.88)	**0.022**
Figure recall (age-adjusted z score), mean (SD)	0.00 (1.00)	0.0422 (0.990)	−0.103 (1.02)	0.178
Verbal memory				
CERAD, immediate recall score trial 1, mean (SD)	4.02 (1.39)	4.24 (1.37)	3.48 (1.30)	**< 0.001**
CERAD, immediate recall score trial 1 (age-adjusted z score), mean (SD)	0.00 (1.00)	0.121 (0.992)	−0.292 (0.961)	**< 0.001**
CERAD, immediate recall score trial 2, mean (SD)	5.97 (1.42)	6.21 (1.41)	5.41 (1.28)	**< 0.001**
CERAD, immediate recall score trial 2 (age-adjusted z score), mean (SD)	0.00 (1.00)	0.109 (1.01)	−0.263 (0.938)	**< 0.001**
CERAD, immediate recall score trial 3, mean (SD)	7.36 (1.38)	7.56 (1.38)	6.87 (1.28)	**< 0.001**
CERAD, immediate recall score trial 3 (age-adjusted z score), mean (SD)	0.00 (1.00)	0.0936 (1.01)	−0.227 (0.947)	**0.002**
CERAD, delay recall score, mean (SD)	5.04 (1.85)	5.36 (1.84)	4.26 (1.62)	**< 0.001**
CERAD, delay recall score (age-adjusted z score), mean (SD)	0.00 (1.00)	0.116 (1.01)	−0.283 (0.913)	**< 0.001**
CERAD, recognition score, mean (SD)	8.97 (1.51)	9.10 (1.39)	8.65 (1.72)	**0.004**
CERAD, recognition score (age-adjusted z score), mean (SD)	0.00 (1.00)	0.0667 (0.927)	−0.163 (1.15)	**0.030**
Executive functioning				
Stroop: word reading, mean (SD)	48.3 (8.12)	48.4 (8.62)	48.2 (6.76)	0.893
Stroop: word reading (age-adjusted z score), mean (SD)	0.00 (1.00)	0.0127 (1.06)	−0.0313 (0.832)	0.708
Stroop: colour reading, mean (SD)	41.1 (6.82)	41.4 (7.04)	40.5 (6.22)	0.254
Stroop: colour reading (age-adjusted z score), mean (SD)	0.00 (1.00)	0.0351 (1.03)	−0.0875 (0.913)	0.299
Stroop: word colour, mean (SD)	45.8 (8.25)	46.0 (8.37)	45.4 (7.95)	0.526
Stroop: word colour (age-adjusted z score), mean (SD)	0.00 (1.00)	0.0315 (1.01)	−0.0783 (0.963)	0.352
Stroop: total, mean (SD)	44.2 (6.25)	44.7 (6.51)	42.8 (5.33)	**0.011**
Stroop: total (age-adjusted z score), mean (SD)	0.00 (1.00)	0.0224 (1.05)	−0.0550 (0.865)	0,513
TMT-B, seconds, mean (SD)	114 (63.5)	107 (60.7)	133 (66.7)	**< 0.001**
TMT-B, seconds (age-adjusted z score), mean (SD)	0.00 (1.00)	−0.0654 (0.959)	0.158 (1.08)	**0.034**
Letter ‘p’, score, mean (SD)	13.4 (4.91)	13.6 (4.96)	12.9 (4.76)	0.149
Letter ‘p’, score (age-adjusted z score), mean (SD)	0.00 (1.00)	0.0195 (1.01)	−0.0471 (0.981)	0.529

Bold value means *p* < 0.05.

**Figure 1:**
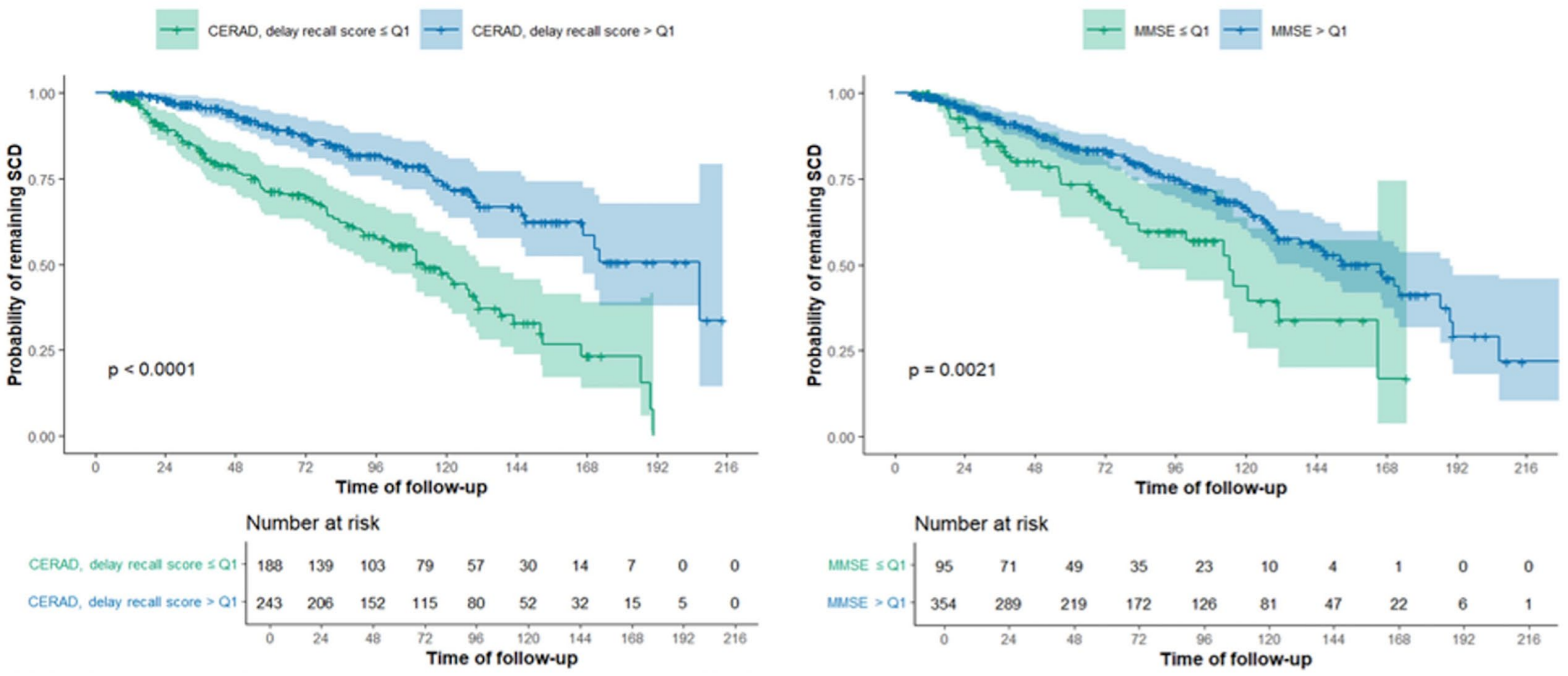
Kaplan–Meier curves for CERAD delayed scores and MMSE, considering the probability of remaining in SCD.

The mean follow-up duration was longer in SCDp patients compared to SCDnp patients (76.33 ± 50.59 months vs. 72.68 ± 51.61 months). Table 2 shows the adjusted risk of progression to MCI or AD. Statistically significant results were found for MMSE (HR = 0.855, IC95% 0.771–0.948, *p* = 0.003), TMT-B (HR = 1.239, IC95% 1.051–1.462, *p* = 0.011), and the CERAD regarding trial 1 of immediate recall (HR = 0.580, IC95% 0.464–0.726, *p* < 0.001), trial 2 of immediate recall (HR = 0.623, IC95% 0.507–0.766, *p* < 0.001), trial 3 of immediate recall (HR = 0.644, IC95% 0.526–0.788, *p* < 0.001) and the delay recall score (HR = 0.657, IC95% 0.532–0.812, *p* < 0.001). Figure 1 shows the probability of remaining in SCD based on the performance on the CERAD delayed recall and the MMSE.

**Table 2:** Association between clinical and neuropsychological variables and the risk of progression.

Variables	HR adjusted (95% CI)	*p* value
Hypertension	1.210 (0.842–1.737)	0.303
Diabetes mellitus	1.097 (0.719–1.673)	0.668
Hypercholesterolemia	0.917 (0.631–1.332)	0.648
Smoking	0.948 (0.635–1.414)	0.793
Cerebrovascular disease	1.031 (0.600–1.773)	0.911
Cardiovascular disease	0.776 (0.420–1.431)	0.416
GDS	0.988 (0.955–1.021)	0.464
MMSE	0.855 (0.771–0.948)	**0.003†**
Processing speed		
TMT-A (z score)	1.144 (0.974–1.35)	0.102
Verbal fluency		
BNT (z score)	0.935 (0.805–1.087)	0.383
Animals (z score)	0.700 (0.663–0.965)	0.020
Constructional ability		
Figure copy (z score)	0.968 (0.868–1.080)	0.561
Visual memory		
Figure recall (z score)	0.837 (0.704–0.995)	0.043
Verbal memory		
CERAD, immediate recall score trial 1 (z score)	0.580 (0.464–0.726)	**< 0.001†**
CERAD, immediate recall score trial 2 (z score)	0.623 (0.507–0.766)	**< 0.001†**
CERAD, immediate recall score trial 3 (z score)	0.644 (0.526–0.788)	**< 0.001†**
CERAD, delay recall score (z score)	0.657 (0.532–0.812)	**< 0.001†**
CERAD, recognition score (z score)	0.805 (0.694–0.934)	0.004
Executive functioning		
Stroop: word reading (z score)	0.944 (0.790–1.128)	0.524
Stroop: colour reading (z score)	0.896 (0.732–1.098)	0.290
Stroop: word colour (z score)	0.893 (0.731–1.090)	0.264
Stroop: total (z score)	0.867 (0.699–1.075)	0.193
TMT-B, seconds (z score)	1.239 (1.051–1.462)	**0.011** ^**†**^
Letter ‘p’, score (z score)	0.867 (0.707–1.063)	0.169

† Adjusted for multiple comparisons. HRs correspond to a one standard deviation (1 SD) increase in standardized cognitive test scores. Bold value means *p* < 0.05.

## 4 Discussion

In this study, we aimed to identify differences between SCDp and SCDnp, as well as variables associated with progression. At baseline, SCDp were older, had a higher prevalence of hypertension and hypercholesterolemia and had worst performance on tests regarding processing speed, verbal fluency, verbal memory, and executive functioning. Factors associated with progression at follow-up were global cognition (MMSE), executive function (TMT-B), and verbal memory test (CERAD regarding trial 1 of immediate recall, trial 2 of immediate recall, trial 3 of immediate recall and the delay recall score).

A limited number of longitudinal studies have explored the prognostic significance of neuropsychological assessments in individuals with SCD regarding their progression to MCI or dementia (Dufouil et al., 2005; Li et al., 2016; Bessi et al., 2018). Evidence suggests the superiority of memory tests, particularly those assessing delayed recall, in predicting the development of dementia in cognitively normal adults. Furthermore, tests measuring executive functions, verbal and visual memory, language, and global cognition are associated with the development of dementia (Vyhnalek et al., 2022).

According to the study carry-out by Miebach et al. (2019), memory and language were the most frequently affected domains in adults with SCD, with executive function impairment observed in less than 10% of cases. Wolfsgruber et al. (2020) supported our findings regarding neuropsychological variables related to SCD. They observed that individuals with SCD performed worse on tests of global cognition, memory, language, and executive functions. Similarly, Bäckman et al. (2005) found similar results in tests of episodic memory, executive functions, and verbal ability. However, they showed that individuals with SCD scored worse on tests of visual ability, attention, and processing speed. More recently, Jester et al. conducted a study that yielded results similar to those presented by Jester et al. (2022).

A cohort study that included both HCs and individuals with SCD found that lower scores on tests of episodic memory, naming, and semantic fluency were associated with an increased risk of progressing to MCI or AD dementia in SCD (Rowe et al., 2010). Hong et al. conducted a study with similar characteristics to determine the risk factors for SCD progression in memory clinics in South Korea (Hong et al., 2015). They performed neuropsychological assessments using the Seoul Neuropsychological Screening Battery. The most relevant risk factors for progression to MCI or AD dementia were poorer results on the MMSE and tests of verbal memory and delayed recall. Additionally, worse results on visual memory tests were associated with a risk of progression, although with less significance (Hong et al., 2015). In a prospective study by Bessi et al. (2018), the predictive role of neuropsychological assessment in the progression to AD dementia from SCD was investigated. The researchers used language and delayed recall test results to construct the Composite Memory Score. They found that lower scores on this test were a significant risk factor for progression to MCI or dementia (Bessi et al., 2018). Several other studies have also used the Composite Memory Score because it has been shown to be highly sensitive in determining the risk of progression from SCD to dementia due to AD (Crane et al., 2012). It is worth noting that this score is the sum of the results of tests of delayed recall and verbal ability.

Our results suggest that vascular risk factors such as hypertension and hypercholesterolemia, are more prevalent in the SCDp group. Both are widely recognized as a well-known risk factor for dementia and accelerates cognitive deterioration in AD through cerebrovascular damage (Ho et al., 2022; Livingston et al., 2024).

This study has limitations that need to be acknowledged. One of the most significant is its retrospective nature, as the data was not originally collected for the purpose of this research. However, this limitation is partially mitigated by the study’s substantial sample size of patients with SCD and the prolonged follow-up period, which allowed for meaningful analyses despite the retrospective design. Another limitation is the loss of subjects during follow-up and the variation in follow-up durations between groups, which could introduce bias or affect generalizability. Nevertheless, the extended tracking of patients over time enabled the correlation of clinical variables both at inclusion and during progression, enhancing the study’s relevance. Furthermore, all participants underwent a complete and standardized neuropsychological assessment, which strengthened the reliability of the cognitive profiles captured and supported the interpretation of the clinical trajectory despite the challenges noted. Finally, although Cox analyses allows for different follow up times, we must acknowledge that duration was longer in SCDp than SCDnp, providinga little more time to develop the outcome.

In future research, conducting a prospective study involving patients with SCD would be advisable, establishing their clinical and neuropsychological profiles during diagnosis and, subsequently, as they progress to MCI and eventually dementia due to AD. Moreover, as we did not include data regarding biomarkers and neuroimaging between progressors and no progressor, future work could also evaluate differences regarding biomarker positivity and structural changes in MRI, as well as functional changes, including frequency dependendent characteristics (Gong and Zuo, 2025).

## 5 Conclusion

Subjects at risk of progressing from SCD to MCI or dementia can be identified through neuropsychological assessments, as poorer performance in specific domains increases progression risk. These findings underscore the importance of considering nuances in neuropsychological evaluation, even if having a normal score, for detecting high-risk individuals aiming at earlier interventions.

## Data Availability

The raw data supporting the conclusions of this article will be made available by the authors, without undue reservation.
