# Supplementary material for: Clinical and neuropsychological features associated with progression in subjective cognitive decline
Source: Front Aging Neurosci. 2025 Nov 26;17:1680762. doi: 10.3389/fnagi.2025.1680762 (PMC12689879; doi:10.3389/fnagi.2025.1680762)
Supplement: Supplementary file 1 [file Table_1.docx]

**Figure 1. Sample diagram.**

**
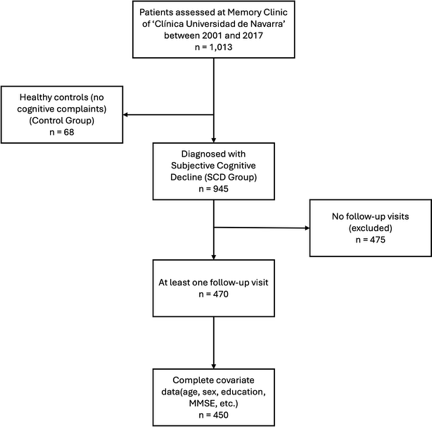
**

**Table 1. Comparison between patients with subjective cognitive decline and healthy controls.**

| **Characteristics** | **Overall** | **SCD** | **Controls** | **p - value** |
| --- | --- | --- | --- | --- |
|  | **(n = 1013)** | **(n = 945)** | **(n = 68)** |  |
| Age, mean (SD) | 64.4 (10.7) | 64.1 (10.9) | 68.6 (7.36) | **< 0.001** |
| Sex, (%) | 502 (49.6%) | 476 (50.4%) | 26 (38.2%) | 0,052 |
| Education in years , mean (SD) | 12.7 (4.19) | 12.9 (4.19) | 10.2 (3.38) | **< 0.001** |
| Hypertension, (%) | 479 (47.3%) | 449 (47.5%) | 30 (44.1%) | 0,590 |
| Diabetes mellitus, (%) | 131 (12.9%) | 131 (13.9%) | 0 (0%) | **< 0.001** |
| Hypercholesterolemia, (%) | 518 (51.1%) | 498 (52.7%) | 20 (29.4%) | **< 0.001** |
| Smoking, (%) | 345 (34.1%) | 335 (35.4%) | 10 (14.7%) | **< 0.001** |
| Cerebrovascular disease, (%) | 51 (5.0%) | 50 (5.3%) | 1 (1.5%) | **0,022** |
| Cardiovascular disease, (%) | 68 (6.7%) | 67 (7.1%) | 1 (1.5%) | **0,001** |
| GDS | 8.78 (5.84) | 9.15 (5.82) | 3.79 (3.36) | **< 0.001** |
| MMSE | 28.5 (1.83) | 28.5 (1.87) | 28.9 (1.24) | **0,004** |
| **Processing speed** |  |  |  |  |
| TMT-A, seconds | 46.2 (22.9) | 45.5 (22.6) | 55.9 (25.1) | **0,002** |
| **Verbal fluency** |  |  |  |  |
| BNT | 51.3 (6.44) | 51.3 (6.49) | 50.1 (5.59) | 0,091 |
| Animals, score | 17.2 (5.40) | 17.0 (5.36) | 19.3 (5.53) | **0,001** |
| **Constructional ability** |  |  |  |  |
| Figure copy, mean (SD) | 9.97 (0.297) | 9.98 (0.252) | 9.85 (0.638) | 0,096 |
| **Visual memory** |  |  |  |  |
| Figure recall, mean (SD) | 6.84 (3.74) | 6.85 (3.74) | 6.73 (3.79) | 0,802 |
| **Verbal memory** |  |  |  |  |
| CERAD, immediate recall score trial 1, mean (SD) | 4.06 (1.35) | 4.09 (1.34) | 3.71 (1.40) | **0,039** |
| CERAD, immediate recall score trial 2, mean (SD) | 6.04 (1.43) | 6.02 (1.44) | 6.26 (1.34) | 0,178 |
| CERAD, immediate recall score trial 3, mean (SD) | 7.41 (1.38) | 7.40 (1.37) | 7.47 (1.53) | 0,724 |
| CERAD, delay recall score, mean (SD) | 5.17 (1.87) | 5.13 (1.85) | 5.64 (1.99) | 0,051 |
| CERAD, recognition score, mean (SD) | 9.05 (1.51) | 9.07 (1.47) | 8.82 (1.99) | 0,317 |
| **Executive functioning** |  |  |  |  |
| Stroop: word reading, mean (SD) | 48.4 (7.75) | 48.4 (7.83) | 48.7 (6.77) | 0,726 |
| Stroop: colour reading, mean (SD) | 41.3 (6.59) | 41.2 (6.54) | 41.6 (7.24) | 0,724 |
| Stroop: word colour, mean (SD) | 45.8 (8.05) | 45.7 (8.09) | 46.5 (7.47) | 0,459 |
| Stroop: total, mean (SD) | 44.2 (6.24) | 44.3 (6.19) | 42.1 (6.58) | **0,012** |
| TMT-B, seconds, mean (SD) | 109 (62.0) | 108 (61.6) | 127 (64.3) | **0,023** |
| Letter ‘p’, score, mean (SD) | 13.8 (5.05) | 13.7 (4.99) | 14.6 (5.77) | 0,229 |

Supplementary table 2. Comparison between the demographic and clinical characteristics of participants included and excluded from the analysis

| **Variables** | **Overall** | **Included** | **Excluded** | **p - value** | **p - value adjusted for multiple comparisons** |
| --- | --- | --- | --- | --- | --- |
|  | **(n = 1013)** | **(n = 450)** | **(n = 563)** |  |  |
| Age, mean (SD) | 64.4 (10.7) | 65.2 (10.2) | 63.8 (11.2) | 0,045 | 0,122 |
| Sex, (%) | 502 (49.6%) | 240 (53.3%) | 262 (46.5%) | 0,032 | 0,095 |
| Education in years , mean (SD) | 12.7 (4.19) | 12.8 (4.23) | 12.7 (4.16) | 0,625 | 0,767 |
| Hypertension, (%) | 479 (47.3%) | 223 (49.6%) | 256 (45.5%) | 0,196 | 0,345 |
| Diabetes mellitus, (%) | 131 (12.9%) | 78 (17.3%) | 53 (9.4%) | **0,000** | **0,002** |
| Hypercholesterolemia, (%) | 518 (51.1%) | 263 (58.4%) | 255 (45.3%) | **0,000** | **0,000** |
| Smoking, (%) | 345 (34.1%) | 144 (32.0%) | 201 (35.7%) | 0,217 | 0,345 |
| Cerebrovascular disease, (%) | 51 (5.0%) | 40 (8.9%) | 11 (2.0%) | **0,000** | **0,000** |
| Cardiovascular disease, (%) | 68 (6.7%) | 41 (9.1%) | 27 (4.8%) | **0,006** | **0,039** |
| GDS | 8.78 (5.84) | 9.10 (5.69) | 8.52 (5.95) | 0,119 | 0,268 |
| MMSE | 28.5 (1.83) | 28.5 (1.57) | 28.5 (2.02) | 0,916 | 0,956 |
| **Processing speed** |  |  |  |  |  |
| TMT-A, seconds | 46.2 (22.9) | 46.1 (19.6) | 46.2 (25.2) | 0,927 | 0,956 |
| **Verbal fluency** |  |  |  |  |  |
| BNT | 51.3 (6.44) | 50.9 (6.75) | 51.5 (6.19) | 0,144 | 0,278 |
| Animals, score | 17.2 (5.40) | 16.6 (5.20) | 17.6 (5.52) | **0,007** | **0,039** |
| **Constructional ability** |  |  |  |  |  |
| Figure copy, mean (SD) | 9.97 (0.297) | 9.98 (0.309) | 9.97 (0.287) | 0,439 | 0,593 |
| **Visual memory** |  |  |  |  |  |
| Figure recall, mean (SD) | 6.84 (3.74) | 6.53 (3.85) | 7.09 (3.64) | 0,022 | 0,085 |
| **Verbal memory** |  |  |  |  |  |
| CERAD, immediate recall score trial 1, mean (SD) | 4.06 (1.35) | 4.02 (1.39) | 4.09 (1.32) | 0,392 | 0,558 |
| CERAD, immediate recall score trial 2, mean (SD) | 6.04 (1.43) | 5.97 (1.42) | 6.09 (1.45) | 0,207 | 0,345 |
| CERAD, immediate recall score trial 3, mean (SD) | 7.41 (1.38) | 7.36 (1.38) | 7.44 (1.38) | 0,345 | 0,517 |
| CERAD, delay recall score, mean (SD) | 5.17 (1.87) | 5.04 (1.85) | 5.27 (1.88) | 0,059 | 0,146 |
| CERAD, recognition score, mean (SD) | 9.05 (1.51) | 8.97 (1.51) | 9.12 (1.51) | 0,137 | 0,278 |
| **Executive functioning** |  |  |  |  |  |
| Stroop: word reading, mean (SD) | 48.4 (7.75) | 48.3 (8.12) | 48.5 (7.47) | 0,673 | 0,790 |
| Stroop: colour reading, mean (SD) | 41.3 (6.59) | 41.1 (6.82) | 41.4 (6.40) | 0,544 | 0,700 |
| Stroop: word colour, mean (SD) | 45.8 (8.05) | 45.8 (8.25) | 45.8 (7.89) | 0,936 | 0,956 |
| Stroop: total, mean (SD) | 44.2 (6.24) | 44.2 (6.25) | 44.2 (6.25) | 0,956 | 0,956 |
| TMT-B, seconds, mean (SD) | 109 (62.0) | 114 (63.5) | 106 (60.5) | **0,031** | **0,095** |
| Letter ‘p’, score, mean (SD) | 13.8 (5.05) | 13.4 (4.91) | 14.1 (5.15) | **0,018** | **0,082** |
